# Argan Oil as an Effective Nutri-Therapeutic Agent in Metabolic Syndrome: A Preclinical Study

**DOI:** 10.3390/ijms18112492

**Published:** 2017-11-22

**Authors:** Adil El Midaoui, Youssef Haddad, Younes Filali-Zegzouti, Réjean Couture

**Affiliations:** 1 Department of Pharmacology and Physiology, Faculty of Medicine, Université de Montréal, Montréal, QC H3C 3J7, Canada; youssef.haddad@umontreal.ca (Y.H.); rejean.couture@umontreal.ca (R.C.); 2 Department of Biology, FST Errachidia, Moulay Ismail University, Errachidia, Morocco; y.filalizegzouti@fste.umi.ac.ma

**Keywords:** metabolic syndrome, oxidative stress, kinin receptors, argan oil

## Abstract

The present study aims at examining the effects of argan oil on the three main cardiovascular risk factors associated with metabolic syndrome (hypertension, insulin resistance and obesity) and on one of its main complications, neuropathic pain. Male Sprague-Dawley rats had free access to a drinking solution containing 10% d-glucose or tap water for 12 weeks. The effect of argan oil was compared to that of corn oil given daily by gavage during 12 weeks in glucose-fed rats. Glucose-fed rats showed increases in systolic blood pressure, epididymal fat, plasma levels of triglycerides, leptin, glucose and insulin, insulin resistance, tactile and cold allodynia in association with a rise in superoxide anion production and NADPH oxidase activity in the thoracic aorta, epididymal fat and gastrocnemius muscle. Glucose-fed rats also showed rises in B_1_ receptor protein expression in aorta and gastrocnemius muscle. Argan oil prevented or significantly reduced all those anomalies with an induction in plasma adiponectin levels. In contrast, the same treatment with corn oil had a positive impact only on triglycerides, leptin, adiponectin and insulin resistance. These data are the first to suggest that argan oil is an effective nutri-therapeutic agent to prevent the cardiovascular risk factors and complications associated with metabolic syndrome.

## 1. Introduction

Metabolic syndrome is an emerging epidemic worldwide that consists of an association of multiple cardiovascular risk factors [1]. These factors, including hypertension, insulin resistance and obesity, directly contribute to a higher incidence of cardiovascular disease and type 2 diabetes. A rise in oxidative stress is one of the main suggested hypotheses to explain the increased risks associated with metabolic syndrome [1,2,3]. Studies have suggested that oxidative stress notably enhanced superoxide anion (O_2_^●−^) production and decreased antioxidant reserve may be implicated in the pathogenesis and complications of hypertension, diabetes and obesity [2,3,4,5,6]. Indeed, studies undertaken in animals have shown that vascular superoxide anion production was elevated in spontaneously hypertensive rats (SHR) [7,8], insulin resistant rats [2] and obese rats [4]. In addition, our previous investigations have shown that vascular NADPH oxidase activity was increased concomitantly with enhanced O_2_^●−^ production in metabolic syndrome rat model induced by 10% d-glucose feeding in the beverage [9,10] and in obese Zucker diabetic fatty rats [11]. In addition, studies have suggested that oxidative stress is implicated in the development of neuropathic pain in hypertensive insulin-resistant rats induced by high glucose feeding [12]. Kinin B_1_ receptor, a biomarker of inflammation, which is virtually absent in healthy tissues, is induced by the cytokine pathway and oxidative stress via the transcriptional nuclear factor κ B (NF-κB) [13]. We have shown that oxidative stress is implicated in the induction and upregulation of the kinin B_1_ receptor in insulin-resistant and hypertensive glucose-fed rats [12] and in obese Zucker diabetic fatty rats [11].

Studies have demonstrated that α-tocopherol (vitamin E) supplementation decreases oxidative stress, lipid peroxidation and the elevated blood pressure in SHR [14,15] and improves insulin action in obese Zucker rats [16] and in type 2 diabetic patients [14,17]. Additionally, investigations have shown that melatonin, a potent antioxidant, exerts beneficial effects on systolic blood pressure, glucose levels, lipid profile and body weight in the fructose-induced metabolic syndrome rat model [18,19]. Melatonin was also found to improve oxidative stress, glucose homeostasis, lipid profile and to reduce body weight gain in young Zucker diabetic fatty rats [20,21,22]. Another potent antioxidant, α-lipoic acid, prevented neuropathic pain and B_1_ receptor upregulation in insulin-resistant hypertensive rats [12] and counteracted the increase in body weight gain by preventing the rise in adipose tissue NADPH oxidase activity and the hepatic upregulation of the kinin B_1_ receptor in obese Zucker diabetic fatty rats [11].

Virgin argan oil is harvested from the fruit of the argan tree (*Argania spinosa* L. Skeels, 1911), which naturally grows in Morocco. Virgin argan oil is obtained by a cold-pressing technique [23] and, consequently, is not altered during the extraction step [24]. Virgin argan oil is characterized by high levels of linoleic and oleic acids, tocopherols [25,26] and melatonin [27,28], which exhibit significant antioxidant activities. It is noteworthy that argan oil is essentially rich in tocopherols particularly in its γ-isoform [25], which is converted to α-isoform in the animal organism [29]. Studies have reported that argan oil-supplemented healthy subjects exhibited lower plasma triglycerides, LDL-cholesterol and total cholesterol levels in association with lower lipid peroxidation and higher α-tocopherol concentrations [30,31,32]. Moreover, investigations have shown that phenolic-extract from argan oil inhibits LDL-cholesterol oxidation in humans. More recently, we have shown that argan oil reduced systolic blood pressure, hyperglycemia and insulin resistance through its antioxidant properties in five-week glucose-fed rats [33]. Argan oil also reduced the increase in plasma triglycerides, total cholesterol and LDL-cholesterol levels, the rise in plasma markers of lipid peroxidation and improved antioxidant status in high fat diet-induced obese rats [34].

To the best of our knowledge, no study has investigated simultaneously the effects of argan oil on the three main cardiovascular risk factors associated with metabolic syndrome (hypertension, insulin resistance and obesity) and on one of its main complications, neuropathic pain. Thus, the present study was designed to investigate whether a chronic treatment of twelve weeks with argan oil can prevent or ameliorate arterial hypertension, insulin resistance, obesity, allodynia, alterations in plasma levels of triglycerides, free fatty acids, leptin and adiponectin in twelve-week glucose-fed rats. The impact of argan oil treatment was also determined on adiposity (number and size of epididymal adipocyte cells), oxidative stress (increase in basal superoxide anion production and NADPH oxidase activity) and on a key marker of inflammation (kinin B_1_ protein expression) in thoracic aorta, gastrocnemius muscle and epididymal fat.

## 2. Results

### 2.1. Blood Pressure

**Figure 1:**
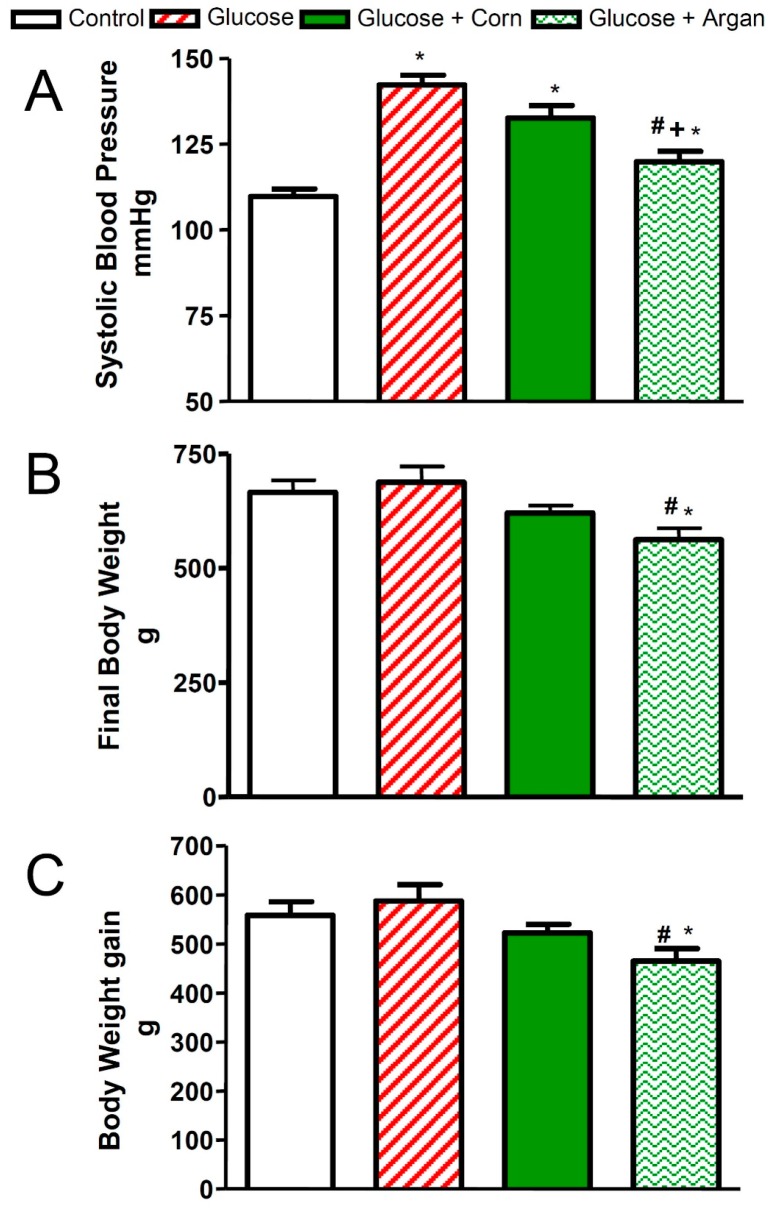
Effects of 12 weeks of glucose feeding combined or not with orally and daily administered (5 mL kg^−1^) corn oil or argan oil on (**A**) systolic blood pressure expressed in mmHg, (**B**) final body weight expressed in g and (**C**) body weight gain expressed in g. Values are the mean ± SEM of 8–10 rats per group. * *p* < 0.05 vs. control, ^#^ *p* < 0.05 vs. glucose, ^+^ *p* < 0.05 vs. glucose + corn oil.

As shown in Figure 1, twelve weeks of treatment with glucose combined or not with corn oil resulted in a significant increase in systolic blood pressure in comparison to control rats. The synchronous feeding with argan oil reduced the rise in systolic blood pressure in glucose-treated rats, so that the systolic blood pressure in argan oil glucose-fed rats was significantly decreased in comparison to glucose or glucose combined with corn oil-fed rats. Chronic glucose feeding combined or not with corn oil had no significant effect on final body weight (Figure 1B) and body weight gain (Figure 1C). However, the chronic treatment with argan oil reduced significantly the final body weight and body weight gain in glucose-fed rats in comparison to control or glucose-fed rats.

### 2.2. Plasma Glucose and Insulin Levels, Insulin Resistance Index and Adiponectinemia

**Figure 2:**
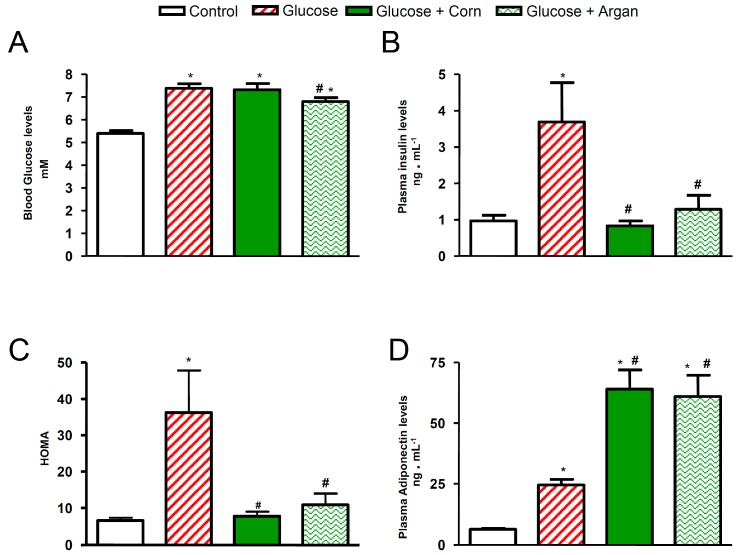
Effects of 12 weeks of glucose feeding combined or not with orally and daily administered (5 mL kg^−1^) corn oil or argan oil on (**A**) blood glucose levels expressed in mM, (**B**) plasma insulin levels expressed in ng mL^−1^, (**C**) HOMA (plasma glucose × insulin/22.5) and (**D**) plasma adiponectin levels expressed in ng mL^−1^. Values are the mean ± SEM of 7–10 rats per group. * *p* < 0.05 vs. control, ^#^ *p* < 0.05 vs. glucose.

As shown in Figure 2A, the twelve weeks of glucose feeding combined or not with corn oil significantly increased the blood glucose levels in comparison to control rats. The chronic treatment with argan oil significantly reduced the rise in blood glucose levels in glucose-fed rats. As shown in Figure 2B, the plasma insulin levels were increased significantly in glucose-fed rats in comparison to control animals. The simultaneous oral feeding with argan oil or corn oil prevented (*p* < 0.05) this increase so that the plasma insulin values of argan and corn oil glucose-fed rats did not statistically differ from that in control rats (Figure 2B). The chronic glucose feeding significantly increased the insulin resistance index, as estimated by HOMA (Figure 2C). The chronic treatment with argan oil or corn oil prevented (*p* < 0.05) this rise so that the insulin resistance index values of argan and corn oil glucose-fed animals did not statistically differ from that in control rats (Figure 2C). As shown in Figure 2D, the twelve weeks of treatment with glucose increased the plasma adiponectin levels by 287% (*p* < 0.05) in comparison to control rats. The synchronous treatment with argan oil or corn oil further increased this parameter by 866% and 915% (*p* < 0.05), respectively, in comparison to control animals (Figure 2D). The plasma adiponectin levels of argan and corn oil glucose-fed rats were significantly higher in comparison to those of rats fed with glucose (Figure 2D).

### 2.3. Epididymal Fat Weight per Body Weight, Lipid Profile and Leptinemia

**Figure 3:**
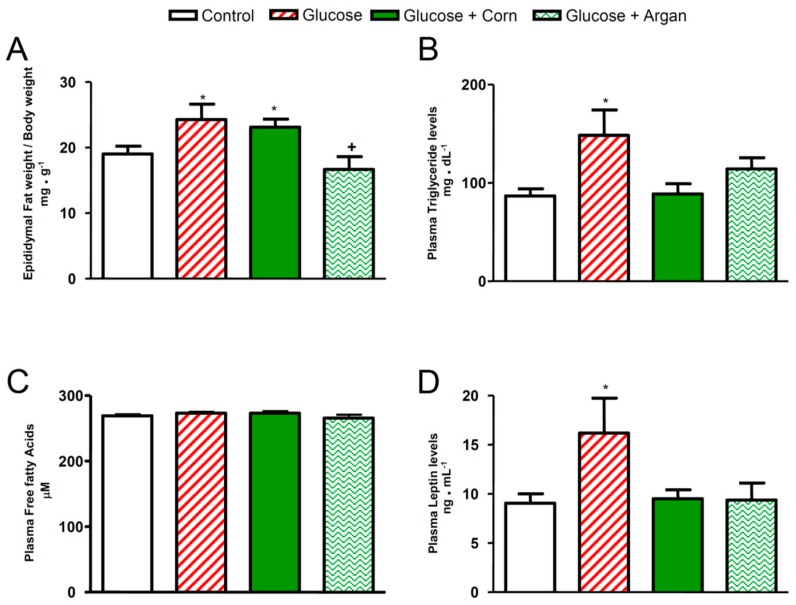
Effects of 12 weeks of glucose feeding combined or not with orally and daily administered (5 mL kg^−1^) corn oil or argan oil on (**A**) epididymal fat weight per body weight expressed in mg g^−1^, (**B**) plasma triglyceride levels expressed in mg dL^−1^, (**C**) plasma free fatty acids expressed in µM and (**D**) plasma leptin levels expressed in ng mL^−1^. Values are the mean ± SEM of 7–10 rats per group. * *p* < 0.05 vs. control, ^+^ *p* < 0.05 vs. glucose + corn oil.

Twelve weeks of treatment with glucose combined or not with corn oil caused a significant increase in epididymal fat weight per body weight in comparison to control rats (Figure 3A). The oral treatment with argan oil prevented the increase in epididymal fat weight per body weight in glucose-fed rats, so that this value did not statistically differ from that in control rats (Figure 3A). As shown in Figure 3B, the plasma triglyceride levels were significantly increased in glucose-fed rats. The simultaneous oral feeding with argan oil or corn oil prevented this increase so that the plasma triglyceride values of argan and corn oil glucose-fed rats did not statistically differ from that in control rats (Figure 3B). As shown in Figure 3C, the plasma free fatty acids’ levels were not significantly modified either by glucose feeding, or by corn oil, or by argan oil. Chronic glucose feeding resulted in a significant increase in plasma leptin levels (Figure 3D). This latter effect was completely prevented by the treatment with argan oil or corn oil. The plasma leptin values of argan and corn oil glucose-fed rats did not statistically differ from that in control rats (Figure 3D).

### 2.4. Number and Size of Epididymal Adipocyte Cells

**Figure 4:**
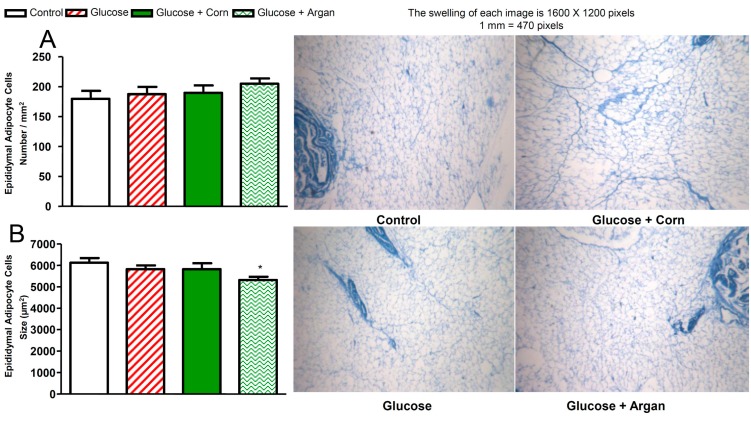
Effects of 12 weeks of glucose feeding combined or not with orally and daily administered (5 mL kg^−1^) corn oil or argan oil on (**A**) epididymal adipocyte cells number expressed in number/mm^2^ and (**B**) epididymal adipocyte cells size expressed in µm^2^. Values are the mean ± SEM of four rats per group.* *p* < 0.05 vs. control. Histology of the adipose tissue pad is also shown for each group.

As shown in Figure 4A, the epididymal adipocyte cells number was not significantly affected either by glucose feeding, or by corn oil, or by argan oil. Moreover, chronic glucose feeding combined or not with corn oil had no effects on epididymal adipocyte cells size (Figure 4B). The twelve weeks of treatment with argan oil, however, significantly decreased the epididymal adipocyte cells size in glucose-fed rats in comparison to control rats (Figure 4B).

### 2.5. Tactile and Cold Allodynia

**Figure 5:**
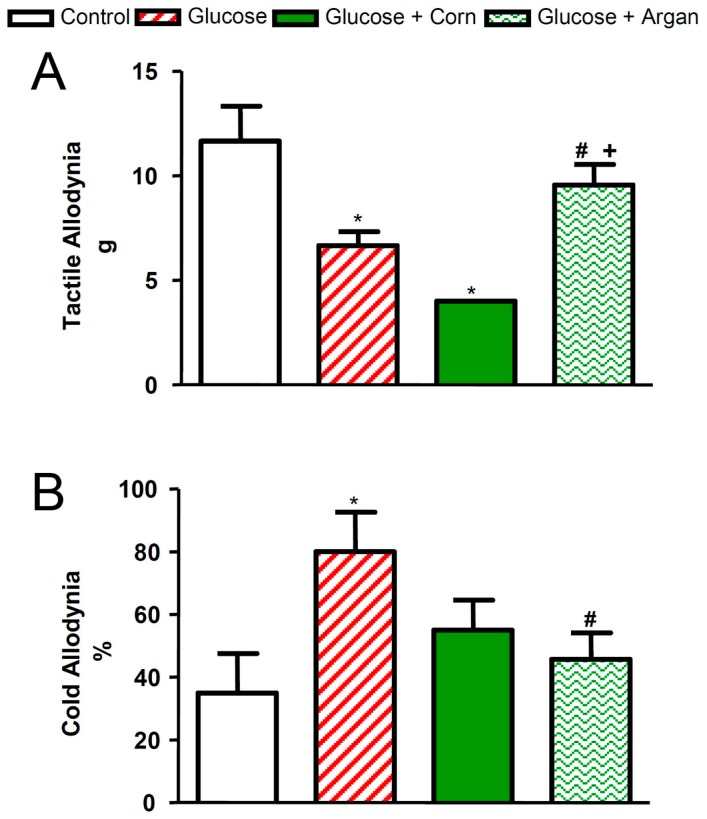
Effects of 12 weeks of glucose feeding combined or not with orally and daily administered (5 mL kg^−1^) corn oil or argan oil on (**A**) tactile allodynia expressed in g and (**B**) cold allodynia expressed in %. Values are the mean ± SEM of 4–7 rats per group. * *p* < 0.05 vs. control, ^#^ *p* < 0.05 vs. glucose, ^+^ *p* < 0.05 vs. glucose + corn oil.

As shown in Figure 5A, the twelve weeks of glucose feeding resulted in significant tactile allodynia in comparison to control rats. Corn oil treatment exacerbated this neuropathic pain syndrome in glucose-fed rats. In contrast, the simultaneous treatment with argan oil prevented (*p* < 0.05) the occurrence of tactile allodynia in glucose-fed rats (Figure 5A). Glucose-fed rats displayed significant cold allodynia when compared to control rats (Figure 5B). Corn oil treatment reduced by 31% this syndrome, although not significantly in glucose-fed rats, while argan oil treatment significantly decreased cold allodynia in glucose-fed rats (Figure 5B). Values of cold allodynia in corn and argan oil glucose-treated rats were not significantly different from control values.

### 2.6. Oxidative Stress Parameters

**Figure 6:**
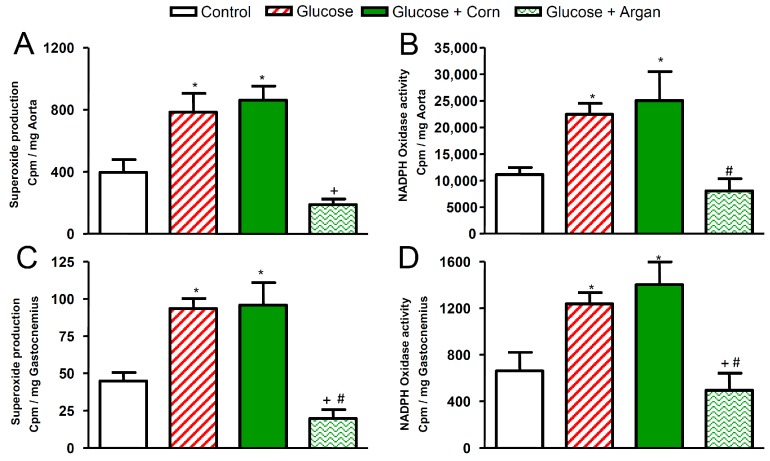

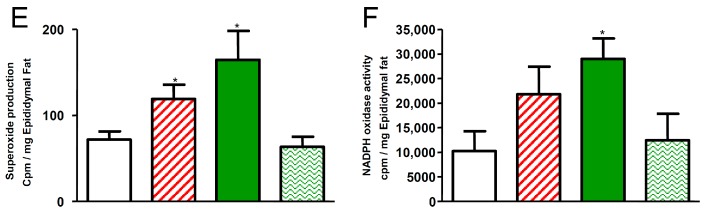
Effects of 12 weeks of glucose feeding combined or not with orally and daily administered (5 mL kg^−1^) corn oil or argan oil on (**A**) superoxide anion production expressed in cpm mg^−1^ of thoracic aorta, (**B**) NADPH oxidase activity expressed in cpm mg^−1^ of thoracic aorta, (**C**) superoxide anion production expressed in cpm mg^−1^ of gastrocnemius muscle, (**D**) NADPH oxidase activity expressed in cpm mg^−1^ of gastrocnemius muscle, (**E**) superoxide anion production expressed in cpm mg^−1^ of epididymal fat and (**F**) NADPH oxidase activity expressed in cpm mg^−1^ of epididymal fat. Values are the mean ± SEM of 4–6 rats per group. * *p* < 0.05 vs. control, ^#^ *p* < 0.05 vs. glucose, ^+^ *p* < 0.05 vs. glucose + corn oil.

The basal superoxide anion production was significantly increased in the thoracic aorta (Figure 6A), gastrocnemius muscle (Figure 6C) and epididymal fat (Figure 6E) of glucose-fed rats and glucose-fed rats co-treated with corn oil. Administration of argan oil prevented this increase in the three tissues of glucose-fed rats, so that the basal superoxide anion production did not significantly differ from that in control rats. Likewise, NADPH oxidase activity was significantly increased in thoracic aorta (Figure 6B), gastrocnemius muscle (Figure 6D) and epididymal fat (Figure 6F) of glucose-fed rats treated or not with corn oil. The simultaneous treatment with argan oil blocked (*p* < 0.05) the rise in NADPH oxidase activity to control values in the three tissues of glucose-fed rats.

### 2.7. Kinin B_1_ Receptor Protein Expression

**Figure 7:**
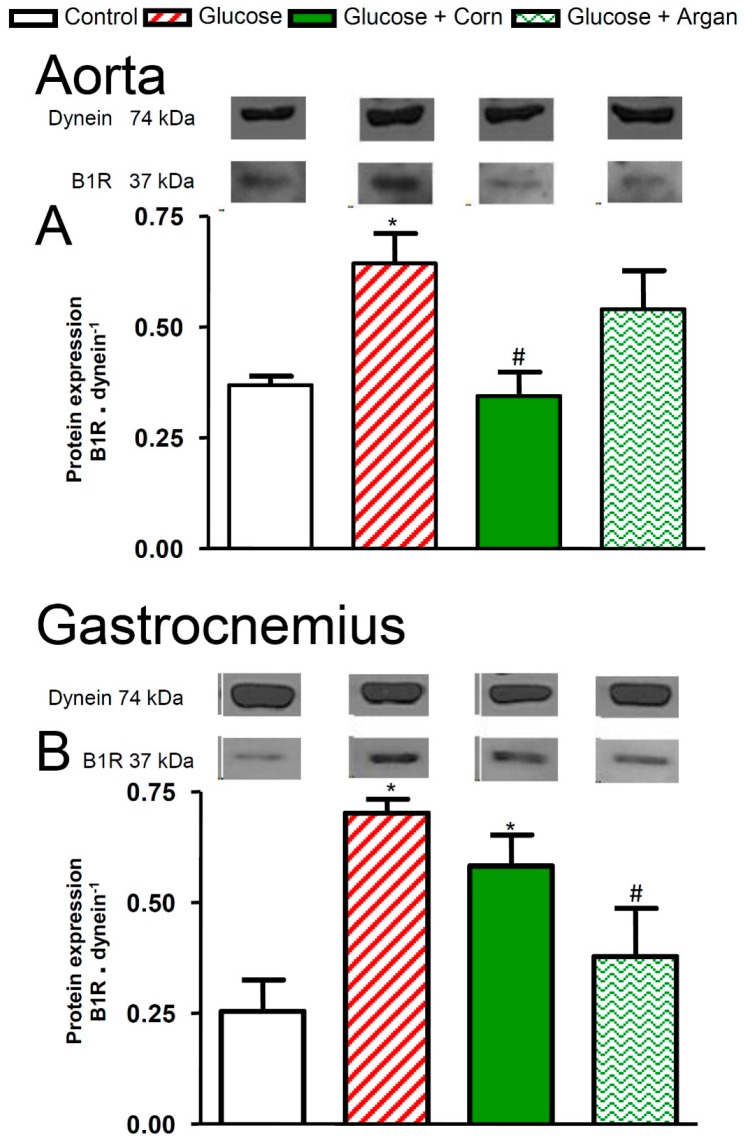
Effects of 12 weeks of glucose feeding combined or not with orally and daily administered (5 mL kg^−1^) corn oil or argan oil on (**A**) kinin B_1_ receptor protein levels in thoracic aorta expressed in B_1_R dynein^−1^ and (**B**) kinin B_1_ receptor protein levels in gastrocnemius muscle expressed in B_1_R dynein^−1^. Values are the mean ± SEM of five rats per group.* *p* < 0.05 vs. control, ^#^ *p* < 0.05 vs. glucose.

As shown in Figure 7A, kinin B_1_ receptor protein expression was significantly increased in thoracic aorta of glucose-fed rats, and this increase was prevented (*p* < 0.05) by corn oil or reduced by argan oil to values not significantly different from the control. Chronic glucose feeding also resulted in a significant increase in B_1_ receptor protein expression in the gastrocnemius muscle (Figure 7B). Treatment with argan oil prevented (*p* < 0.05) the rise in B_1_ receptor protein expression in glucose-fed rats to values that did not significantly differ from control (Figure 7B). In contrast, corn oil failed to affect this rise in the gastrocnemius muscle in glucose-fed rats significantly (Figure 7B). B_1_ receptor protein expression was not detectable in the epididymal fat of the four groups of rats.

## 3. Discussion

This study showed enhanced basal superoxide anion production and NADPH oxidase activity in epididymal fat, gastrocnemius muscle and thoracic aorta in association with an upregulation of kinin B_1_ receptor protein expression in gastrocnemius muscle in rats treated for 12 weeks with glucose combined or not with corn oil. This was accompanied by an increase in epididymal fat weight per body weight, plasma levels of triglycerides and leptin together with the development of hyperglycemia, hyperinsulinemia, insulin resistance, hypertension and allodynia, a feature of neuropathic pain. Importantly, all these metabolic syndrome features were prevented or reduced by argan oil feeding.

Data on blood pressure are consistent with previous studies in which we have shown that oral glucose feeding for three, four or five weeks caused an increase of systolic blood pressure [33,35,36]. Since high blood pressure and enhanced basal superoxide anion production and NADPH oxidase activity from aortic tissue in glucose-fed rats were not affected by corn oil, this may suggest an implication of the vascular oxidative stress in the sustained elevation of systolic arterial pressure induced by chronic glucose feeding. This is in accordance with previous studies, which have shown an antihypertensive effect of argan oil in obese insulin-resistant rats [37], SHR [38] and five-week glucose-fed rats [33]. This is in keeping with the beneficial effect of argan oil supplementation on plasma lipid profile and oxidant-antioxidant status in the rat model of high-fat diet-induced obesity [34]. Argan oil was found to be rich in compounds with significant antioxidant activities particularly tocopherols [25,26] and melatonin [27,28]. Interestingly, antioxidant treatment with α-tocopherol was found to reduce oxidative stress and the increase in blood pressure in SHR [15]. Moreover, investigations have reported that phenolic extract from argan oil inhibits human low-density lipoprotein (LDL) oxidation [39]. Additionally, melatonin counteracted the increase in systolic blood pressure in the fructose-induced metabolic syndrome rat model [19]. Therefore, the present study suggests that argan oil, which is rich in antioxidant compounds notably tocopherols, melatonin and sterols, reduced blood pressure through the prevention of oxidative stress that may also involve kinin B_1_ receptor upregulation at the vascular level in chronically-glucose-fed rats. To support this hypothesis, kinin B_1_ receptor antagonism and the use of other antioxidant therapies (α-lipoic acid, *N*-Acetyl-l-cysteine) reversed arterial hypertension together with the oxidative stress and B_1_ receptor upregulation in this model of metabolic syndrome [12,40,41].

In the present study, the preventive effect of argan oil on hyperinsulinemia and insulin resistance might be associated with the blockade of the kinin B_1_ receptor protein overexpression in the gastrocnemius muscle and its composition in fatty acids notably in linoleic and oleic acids [42] in glucose-fed rats. This is in accordance with a previous study [34], which has reported that four weeks of treatment with argan oil blunted hyperglycemia and hyperinsulinemia in obese insulin-resistant rats. Argan oil normalized blood glucose levels and improved insulin sensitivity in fat and liver tissues of obese insulin-resistant rats [43]. α-tocopherol, among the main compounds found in argan oil, improved insulin action and reduced plasma lipid peroxidation in obese insulin-resistant Zucker rats [16]. Additionally, melatonin, another compound of argan oil, was found to improve oxidative stress and glucose homeostasis in young Zucker diabetic fatty rats [21,22], although it remains uncertain whether the low quantity of melatonin in argan oil (60.5 ng·kg oil^−1^) contributes to the beneficial effects on oxidative stress and glucose metabolism in glucose-fed rats. The beneficial effect of corn oil on hyperinsulinemia and insulin resistance in glucose-fed rats is in agreement with our recent study in five-week glucose-fed rats [33] and with previous studies that have reported that oleic and linoleic acids, the principal compounds of corn oil [44], improved insulin sensitivity in skeletal muscle cells [45].

Argan oil treatment resulted in a high increase in plasma adiponectin levels in chronically-glucose-fed rats. Adiponectin, a protein secreted by adipocytes, increases the sensitivity of insulin by activating muscle glucose uptake via promoting Glut 4 translocation and inhibiting hepatic gluconeogenesis [46]. Adiponectin was also found to act as an agonist of peroxisome proliferator activated receptor γ (PPARγ), leading to additional plasmatic glucose uptake [46]. PPARγ exerts an inhibitory effect on NF-κB, a nuclear factor involved in transcription of many genes encoding inflammatory proteins [47] such as the kinin B_1_ receptor [13]. Thus, the inhibition of NF-κB by the marked increase of adiponectin levels in rats fed with argan oil may account for the suppression of kinin B_1_ receptor expression in glucose-fed rats. Therefore, the present study suggests that argan oil prevents the development of hyperinsulinemia and insulin resistance by counteracting the kinin B_1_ receptor upregulation in skeletal muscle and by increasing plasma adiponectin levels independently of an action on oxidative stress in chronically-glucose-fed rats.

Furukawa et al. [48] have suggested that increased oxidative stress in accumulated fat is an important pathogenic mechanism of obesity-induced metabolic syndrome. Argan oil prevented the increase in epididymal fat weight and plasma triglyceride levels and the rise in superoxide production and NADPH oxidase activity in the epididymal fat of glucose-fed rats. This is in agreement with a previous study [34], which has reported that a diet supplemented with argan oil reduced the increase in adipose tissue weight, body weight and serum triglyceride concentrations in high fat diet-fed rats. Treatment with NADPH oxidase inhibitor reduced ROS production in adipose tissue, improved hypertriglyceridemia, hyperglycemia, hyperinsulinemia and adiponectin levels in obese mice [48]. Studies have reported that adiponectin inhibits the synthesis of fatty acids and stimulates their oxidation [46]. These findings are in accordance with our results showing a reducing effect of argan oil on adipocyte size, fat pads and body weight in chronically-glucose-fed rats. Hence, the lower body weight could be responsible for the improvement of the metabolic parameter, notably plasma triglyceride levels in argan oil glucose-fed rats. Moreover, the fact that argan oil and NADPH oxidase inhibitor exert comparable beneficial effects on body weight, plasma levels of triglycerides, glucose, insulin and adiponectin suggest that adipose tissue oxidative stress is implicated in the visceral obesity and insulin resistance observed in the chronically-glucose-fed rat model.

Treatment with glucose caused a significant increase in plasma leptin levels. These results are in accordance with previous studies showing elevated serum leptin levels in this model of glucose-fed rats [49] and in obese rats and humans [34,50]. Studies have suggested that the obese state is characterized by leptin resistance [50]. Interestingly, argan oil prevented the increase in plasma leptin levels in chronically-glucose-fed rats. This may suggest that argan oil increases the sensitivity to leptin in the hypothalamus. Sour et al. [34] did not observe any effect of supplemented diet with argan oil on the rise in plasma leptin levels in high fat-treated rats. This discrepancy may be explained by the difference in the animal model of metabolic syndrome, the dose and the duration of argan oil treatment used between the two studies. Melatonin was shown to decrease plasma leptin levels and body weight in obese rats [18,51], and α-tocopherol decreased plasma triglyceride levels in obese high fat-treated animals [52,53]. Nevertheless, the fact that corn oil prevented the rise in plasma leptin levels with no change in superoxide anion production and NADPH oxidase activity in adipose tissue suggests that argan oil exerts its beneficial effect on leptin levels through a mechanism unrelated to oxidative stress. The present study also showed that argan oil prevented tactile and cold allodynia in chronically-glucose-fed rats. This is in agreement with previous studies [12,40], which have shown that α-lipoic acid and *N*-acetyl-l-cysteine, two potent antioxidants, alleviated tactile and cold allodynia in glucose-fed rats. Therefore, one may suggest that argan oil exerts its beneficial effects on sensory abnormalities associated with metabolic syndrome through its blockade of oxidative stress. Indeed, corn oil, which was devoid of antioxidant properties, exacerbated tactile allodynia and was less effective than argan oil at reducing cold allodynia.

## 4. Materials and Methods

### 4.1. Animals and Protocols

All research methods and animal care procedures were approved by the Animal Care Committee of the Université de Montréal (Protocol 15-084; Approval date: 3/09/ 2015) in compliance with the guiding principles as enunciated by the Canadian Council on Animal Care and the ARRIVE guidelines [54,55]. Male Sprague-Dawley (SD) rats weighing 70–80 g (Charles River Laboratories, St-Constant, QC, Canada) were housed two per cage, under controlled conditions of temperature (22 °C) and humidity (43%), on a 12-h light-dark cycle. One week after their arrival, rats were randomly divided into four groups of 10 rats and treated for 12 weeks as follows: Group 1 had free access to a drinking solution of 10% d-glucose (Sigma-Aldrich, Oakville, ON, Canada) and to a standard chow diet (2018 Teklad Global 18% Protein Rodent Diet); Group 2 had free access to 10% d-glucose and was fed daily by gavage with argan oil (5 mL kg^−1^); Group 3 had free access to 10% d-glucose and was fed daily by gavage with corn oil (5 mL kg^−1^); Group 4, represents the control group and had free access to tap water only and to the same standard chow diet. It is noteworthy that animals were handled every day, one week before the beginning of gavage and through the period of treatment. Moreover, our laboratory staff is well trained with the gavage procedure that we used in previous articles for other treatments [33,41]. Therefore, we did not feel at any time that animals were stressed by the gavage procedure. Argan oil was obtained from Argan3 Inc. (Montreal, QC, Canada) and was 100% pure with the following composition: palmitic acid 14%, stearic acid 5%, oleic acid 43.5%, linoleic acid 37%, linolenic acid 0.6%, sterols, schottenol 45%, spinasterol 35% and tocopherols 1034 mg·kg^−1^ with no additives or ingredients. A recent study has shown that virgin argan oil also contains melatonin (60.5 ng kg oil^−1^), which is a potent antioxidant [27]. The duration of treatment with argan oil was based on our previous investigations showing that fourteen weeks of treatment with the antioxidant α-lipoic acid prevented the development of arterial hypertension, insulin resistance and reduced the plasma lipid levels in glucose-fed rats [9]. Corn oil was purchased from ACH Food Companies Inc. (Oakville, ON, Canada) and has the following composition: palmitic acid 13%, stearic acid 3%, oleic acid 31%, linoleic acid 52% and linolenic acid 1%. Corn oil was found to be safe with no clinical signs or toxicity at 5 mL kg^−1^ day^−1^ for 12 weeks in treated SD rats [56]. We compared corn oil to argan oil because they contain approximately the same composition of fatty acids with the exception that argan oil also contains tocopherols, sterols and, to lesser extent, melatonin [42]. The dose of argan and corn oil was selected on the basis of our previous study [33], which failed to show any negative effects when administered for a period of 5 weeks in this model and on the basis of other studies using 5 mL kg^−1^ day^−1^ chronically administered by oral gavage in other animal models [37]. If one takes into consideration differences in the body surface area between rat and human, the dose of 5 mL kg^−1^ day^−1^ has to be divided by 6.2 to achieve the equivalent dose in human [57]. This dose is comparable with doses used in human studies [58].

After 12 weeks of treatment, systolic blood pressure was measured by tail-cuff plethysmography based on an average of 5 readings per animal (ADI Instruments Inc., Colorado, CO, USA) and registered with the ADI Instruments Program (Lab Chart Pro7.Ink). The rats were sacrificed by decapitation after light anesthesia with isoflurane. The blood was collected in a vacutainer tube early in the morning after fasting overnight (16 h) for the subsequent measurements of plasma insulin, triglycerides, free fatty acids, leptin and adiponectin. Three representative tissues involved in hypertension, insulin sensitivity and visceral obesity (thoracic aorta, gastrocnemius muscle and epididymal fat) were removed and kept frozen at −20 °C until the subsequent measurement of oxidative stress and kinin B_1_ receptor expression.

### 4.2. Measurement of Metabolic Parameters

Blood glucose concentrations in overnight-fasted rats were measured with a glucometer (Accu-Chek Aviva, Roche Diagnostics, Laval, QC, Canada). Plasma insulin, adiponectin and leptin were measured by Rat/Mouse ELISA kits from Millipore Canada Limited (Etobiocoke, ON, Canada). Plasma triglycerides and free fatty acids were measured by enzymatic kits from Cayman Chemical Company (Ann Arbor, MI, USA). To evaluate the degree of insulin resistance, the Homeostasis Model Assessment (HOMA) was used as an index of insulin resistance and calculated by the following formula: insulin (µU mL^−1^) × glucose (mM) ÷ 22.5 [59].

### 4.3. Measurement of Allodynia

Tactile and cold allodynia were assessed with the rats placed on a wire mesh floor beneath an inverted plastic cage. The rats were allowed to adapt for about 15 min or until explorative behaviour ceased. Tactile allodynia was assessed by measuring the hind paw withdrawal threshold to the application of a calibrated series of six von Frey filaments (bending forces of 2, 4, 6, 8, 10 and 15 g) applied perpendicularly to the mid-plantar surface as described previously [12,40,60]. Cold allodynia was assessed using the acetone drop method applied to the plantar surface of the hind paws as previously described [12,40,60]. The frequency of paw withdrawal was expressed as a percentage (the number of paw withdrawals ÷ number of trials × 100).

### 4.4. Measurement of Superoxide Anion and NADPH Oxidase Activity

Superoxide anion (O_2_^●−^) production was measured from frozen thoracic aorta, gastrocnemius muscle and epididymal fat using the lucigenin-enhanced chemiluminescence method as described previously [11,61,62]. Briefly, small slices of tissues were preincubated in Krebs-HEPES buffer (saturated with 95% O_2_ and 5% CO_2_, at room temperature) for 30 min and then transferred to a glass scintillation vial containing 5 µM of lucigenin for the determination of basal O_2_^●−^ levels. The chemiluminescence was recorded every minute for 10 min at room temperature in a liquid scintillation counter (Wallac 1409, Turku, Finland). Lucigenin counts were expressed as cpm mg^−1^ of dry weight tissue. Moreover, NADPH oxidase activity in the samples was assessed by adding 0.1 mM NADPH to the vials before counting [63]. The background was counted using a vial containing the solution with no tissue. The basal superoxide production was evaluated by subtracting the background count from the luminescence value induced by the addition of tissue slices while the NADPH oxidase activity was measured by subtracting the background count from the luminescence value induced by the addition of tissue slices plus NADPH.

### 4.5. Western Blot Analysis

Western blot was performed as described previously [49]. Total proteins were loaded (20–30 μg) in each well of 10% SDS-PAGE. Dynein was used as the standard protein. Detection of kinin B_1_R was made with a specific polyclonal rabbit antiserum (1:1000) [64,65]. Detection of dynein was made with anti-dynein antibody (1:4000 mouse, SC-13524; Santa Cruz Biotechnology, Santa Cruz, CA, USA). Secondary antibodies were horseradish peroxidase (HRP)-linked goat anti-rabbit SC-2004 and HRP-linked goat anti-mouse SC-2005 (Santa Cruz) used at a dilution of 1:25,000 (for B_1_R) and 1:5000 (for dynein). A quantitative analysis of proteins was provided by scanning densitometry using the MCID-M1 system (Imaging Research, St. Catharines, ON, Canada).

### 4.6. Adipocyte Morphometry

After overnight fixation in 4% paraformaldehyde, epididymal fat wedges were embedded in paraffin and cut into 20-μm sections. Slides were stained with methylene blue after deparaffinisation. Sections snapshots, taken using a DAGE-MTI CCD72 digital camera, were analyzed with MCID-M1 software (Imaging Research). Adipocyte cells size, expressed in µm^2^, represents the average of 20 cells per section in 5 sections per animal for 5–6 rats per group. Adipocyte cells number, expressed as the number per mm^2^, represents the average of 20–30 mm^2^ per section in 5 sections per animal for 5–6 rats per group. Color photomicrographs were taken with a camera (QImaging Retiga-2000R, Burnaby, BC, Canada).

### 4.7. Statistical Analysis of Data

Data are expressed as the mean ± SEM of values obtained from *n* rats in each group. Statistical analysis was performed using Prism^TM^ Version 5.0 (GraphPad Software Inc., La Jolla, CA, USA); data and statistical analysis comply with the recommendations on experimental design and analysis in pharmacology [66]. Data were subjected to one-way ANOVA, followed by the Bonferroni/Dunn multiple comparison test when F achieved *p* < 0.05, and there was no significant variance in homogeneity. Significance was considered when *p* < 0.05.

## 5. Conclusions

Data show that argan oil prevented the increase in visceral obesity, plasma triglyceride levels, hyperinsulinemia, insulin resistance, neuropathic pain and reduced hyperglycemia and high blood pressure in a model of metabolic syndrome induced by high glucose feeding. These beneficial effects of argan oil are probably explained, at least in part, by (1) improvement of the metabolic status due to the loss of body weight, (2) its antioxidant properties, (3) the suppression of kinin B_1_ receptor upregulation and (4) the consequent inhibition of hyperleptinemia and the increase of plasma adiponectin levels. Thus, the present study supports the use of argan oil as a potential nutri-therapeutic agent in the prevention of hypertension, insulin resistance and obesity.

## Abbreviations

BK	Bradykinin
GLUT 4	Glucose transporter 4
HOMA	Homeostasis model assessment
NF-κB	Transcriptional nuclear factor κ B
NADPH	Nicotinamide adenine dinucleotide phosphate hydrogen
O_2_^●−^	Superoxide anion
PPARγ	Peroxisome proliferator activated receptor γ
SHR	Spontaneously hypertensive rat
